# Designing a Framework for Remote Cancer Care Through Community Co-design: Participatory Development Study

**DOI:** 10.2196/29492

**Published:** 2022-04-12

**Authors:** Eliah Aronoff-Spencer, Melanie McComsey, Ming-Yuan Chih, Alexandra Hubenko, Corey Baker, John Kim, David K Ahern, Michael Christopher Gibbons, Joseph A Cafazzo, Pia Nyakairu, Robin C Vanderpool, Timothy W Mullett, Bradford W Hesse

**Affiliations:** 1 Design Lab University of California San Diego La Jolla, CA United States; 2 Division of Infectious Diseases and Global Public Health Department of Medicine UC San Diego School of Medicine La Jolla, CA United States; 3 Department of Health & Clinical Sciences College of Health Sciences University of Kentuck Lexington, CA United States; 4 Qualcomm Institute University of California San Diego La Jolla, CA United States; 5 Department of Computer Science College of Engineering University of Kentucky Lexington, KY United States; 6 Department of Psychiatry Brigham and Women’s Hospital Boston, MA United States; 7 Greystone Group, Inc Washington, DC United States; 8 University Health Network Toronto, ON Canada; 9 National Cancer Institute Bethesda, CA United States; 10 National Cancer Institute (Retired) Kona, HI United States

**Keywords:** cancer care, distress screening, human-centered design, participatory design, Appalachia, mobile phone

## Abstract

**Background:**

Recent shifts to telemedicine and remote patient monitoring demonstrate the potential for new technology to transform health systems; yet, methods to design for inclusion and resilience are lacking.

**Objective:**

The aim of this study is to design and implement a participatory framework to produce effective health care solutions through co-design with diverse stakeholders.

**Methods:**

We developed a design framework to cocreate solutions to locally prioritized health and communication problems focused on cancer care. The framework is premised on the framing and discovery of problems through community engagement and lead-user innovation with the hypothesis that diversity and inclusion in the co-design process generate more innovative and resilient solutions. Discovery, design, and development were implemented through structured phases with *design studios* at various locations in urban and rural Kentucky, including Appalachia, each building from prior work. In the final design studio, working prototypes were developed and tested. Outputs were assessed using the System Usability Scale as well as semistructured user feedback.

**Results:**

We co-designed, developed, and tested a mobile app (myPath) and service model for distress surveillance and cancer care coordination following the *LAUNCH (Linking and Amplifying User-Centered Networks through Connected Health)* framework. The problem of awareness, navigation, and communication through cancer care was selected by the community after framing areas for opportunity based on significant geographic disparities in cancer and health burden resource and broadband access. The codeveloped digital myPath app showed the highest perceived combined usability (mean 81.9, SD 15.2) compared with the current gold standard of distress management for patients with cancer, the paper-based National Comprehensive Cancer Network Distress Thermometer (mean 74.2, SD 15.8). Testing of the System Usability Scale subscales showed that the myPath app had significantly better usability than the paper Distress Thermometer (t_63_=2.611; *P*=.01), whereas learnability did not differ between the instruments (t_63_=–0.311; *P*=.76). Notable differences by patient and provider scoring and feedback were found.

**Conclusions:**

Participatory problem definition and community-based co-design, *design-with* methods, may produce more acceptable and effective solutions than traditional *design-for* approaches.

## Introduction

### Background

The COVID-19 pandemic has upended the US health care delivery system, putting a spotlight on long-standing health and economic inequities, gaps in care, uneven quality, and fragmentation in service models [1]. The rapid implementation and uptake of telemedicine and virtual care in response to the pandemic has been a positive shift [2-4]; however, existing flaws in the system remain, and new challenges to reliable system integration with virtual care are likely, especially in rural regions [5]. The impact of the pandemic on cancer care is particularly worrisome because screening and diagnostic testing have been significantly curtailed or delayed, resulting in projections of a substantial rise in excess deaths from cancer [6]. Of particular concern, patients have been reluctant to return to health care facilities out of concern for exposure to the novel coronavirus.

These compounding challenges have exposed an imperative to redesign systems of cancer care that are “anti-fragile” [7]: resilient, flexible, and democratic. In particular, techniques associated with human-centered design (HCD), such as participatory design; community engagement; and the iterative, collaborative development of sustainable solutions, have been considered essential in re-establishing trust in a health care system challenged during the pandemic [8]. In the tradition of HCD proponents such as von Hippel [9], we believe that innovation to solve problems requires working with the very people, in their own context, whom the problem affects. These are the people who understand the problem best, for whom the solutions must work, and who possess critical knowledge about local resources or potential obstacles to success.

### This Study

Drawing on recommendations from a 2016 report issued by the legislatively mandated *President’s Cancer Panel* [10] and in the spirit of the *Beau Biden Cancer Moonshot*, this study describes a human-centered participatory design approach to engage patient, caregiver, and community stakeholders in solving a community-defined problem: here, the problem of serving rural patients experiencing distress during cancer treatment. The effort represents the work of an interagency, public–private partnership called the *LAUNCH (Linking and Amplifying User-Centered Networks through Connected Health)* initiative [11]. As a demonstration project, this study illustrates not only the richness and creativity that can come from co-design methods, but also their potential efficacy: our co-designed intervention outperformed the standard of care in usability testing.

For the demonstration project, Appalachian Kentucky was identified as a region that could benefit most from this kind of participatory design because of both positive and negative attributes: high cancer burdens, connectivity challenges related to rural geography, higher poverty rates, increased social capital, and a historical tradition of community engagement and resource sharing [12]. According to data from the National Program of Cancer Registries, cancer incidence [13] and mortality [14] in Appalachia are among the highest in the nation, and differences between counties and some unincorporated areas can show even starker disparities. Appalachia is also home to some of the most rural and difficult-to-access communities in the United States. Although these communities may benefit most from telemedicine and remote care, broadband access and adoption remain among the lowest in the country [15,16].

During the ethnographic research conducted before this co-design project, we developed relationships with community leaders in Appalachian Kentucky and identified communities where similar initiatives have had success because of high interest and engagement. Our ethnographic work demonstrated that community service and collaborative problem solving are a regular part of the social fabric of many Appalachian communities [12]. Our ethnographic work also demonstrated that more top-down approaches to systemic innovation were likely to fail in the region because of historic exploitation of the people and the land by outsiders [17].

In this context, we adopted a formal design process with roots in HCD, which is used to innovate in complex sociotechnical systems such as health care [18]. This approach may also be referred to as DesignX [19], co-design [20], or participatory design [21], each of which has been shown to improve the efficacy, adoption, and trust of new health services [8]. This approach is consistent with, and an extension of, scenario-based design proposed by Carroll [22] and borrows heavily from rapid contextual design tools that are well reviewed by Holtzblatt et al [23]. Throughout this paper, we treat co-design and cocreation as related but distinct concepts. Co-design is a set of techniques that engage all relevant stakeholders in the formulation, design, and iterative testing of ≥1 solutions to a given problem. Cocreation is the overall process of bringing together stakeholder communities to enable development of co-designed solutions, which are implemented within, and used by, these stakeholder communities.

In what follows, we describe the participatory design, development, and usability testing of a mobile app initially framed as a system to facilitate communication about cancer-related distress among patients, caregivers, and providers. We situate these processes within a novel framework we refer to as the LAUNCH Roadmap. We then present results from formative evaluation of the app and service model.

## Methods

### People and Context

We first engaged stakeholders in Kentucky through ethnographic methods described in the study by McComsey et al [12]. These included semistructured interviews and in situ observations with patients with cancer and cancer survivors; family and caregivers; and providers, payers, technologists, and broadband providers. The purpose of the ethnographic research was to understand the experience of having cancer in Appalachia, to take inventory of the health care and connectivity resources supporting cancer care in rural Kentucky, and to develop a network of local champions to continue similar projects of inquiry and co-design.

### The Participatory Design Process

**Figure 1:**
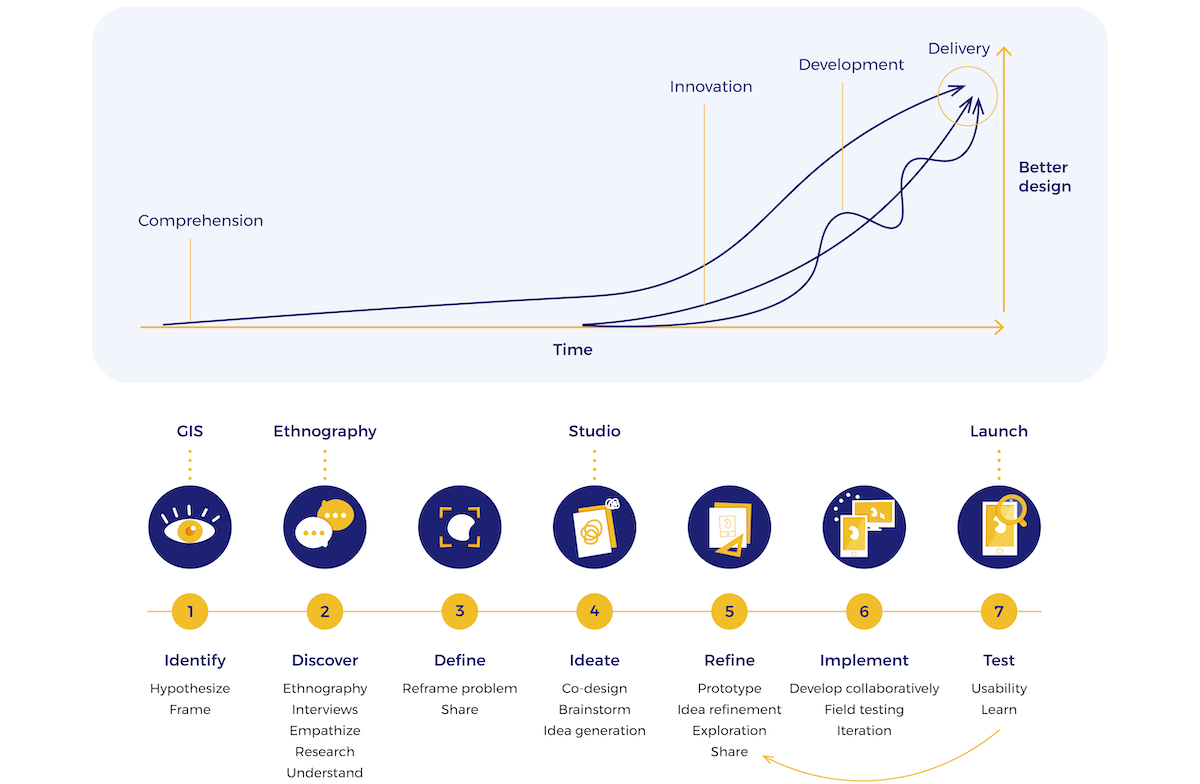
LAUNCH (Linking and Amplifying User-Centered Networks through Connected Health) Roadmap. Visual journey of the co-design process: From problem identification to delivery: Above: Co-design assumes that problem comprehension, innovation, and development codevelop through a process of participatory iteration. Below: The 7 recursive stages that begin with problem identification, discovery, and definition, followed by ideation, prototyping, and refinement through deployment.

Our participatory design process included deliberate opportunities for innovation and iteration of the process itself. This report details the participatory design process as it emerged during collaborative problem solving. The *LAUNCH Roadmap* combines both novel and proven approaches to participatory design. This framework is detailed in prior work by the LAUNCH initiative [11,12] and summarized in this paper in Figure 1.

According to the LAUNCH Roadmap, the innovation process begins with (1) problem identification, which is an iterative process requiring (2) contextual discovery and (3) collaborative problem defining. Next, (4) an ideation step generates potential solutions, which are further (5) refined and ultimately (6) implemented and (7) tested (Figure 1). Our process culminated in a systematic comparison of our outcomes with standard of care outcomes, a project that is underway at the time of this writing.

The LAUNCH Roadmap itself was emergent throughout our process, rather than being an existing framework to be applied (Table 1). We see the LAUNCH Roadmap as a blueprint that can be adapted to different contexts and that may emerge in different forms when iterated upon within the course of any community co-design project. As design processes must be tailored to local contexts, frameworks for implementation must be flexible and follow a bottom-up trajectory of emergence.

**Table 1:** LAUNCH (Linking and Amplifying User-Centered Networks through Connected Health) Roadmap activities (identify, discover, define, ideate, refine, implement, and test).

Purpose			Location (date)		Activity		Outcome	
**Identify**								
	Identify the intersection between connectivity and cancer	Lexington, Kentucky (November 4, 2017) La Jolla, California (March 3, 2018)		LAUNCH kickoff Quantitative and GIS^a^ presentation on the double burden of cancer and connectivity				Formalized interagency partnership; executed memorandum of understanding between FCC^b^ and NCI^c^ to share complementary technical and policy expertise through C2H^d^
	Engage local stakeholders	Kentucky (June-September 2018)		Local stakeholder meetings				Recruited coalition of local champions, connectivity stakeholders, and friends of LAUNCH
	Engage national-level stakeholders	Washington, DC (May 5, 2019)		Information gathering from industry, government, and academic experts				Graphical documentation of stakeholder data and perspectives Meeting summary: L.A.U.N.C.H. Senior Leadership Think Tank: Exploring the Future of Connected Cancer Care in Rural America and Beyond
**Discover**								
	Understand the experiences of patients with cancer, cancer survivors, caregivers, and health and broadband providers. Document the cancer care and connectivity infrastructure available in eastern Kentucky	Kentucky (June 2018 to September 2018)		Ethnographic interviews Contextual observation Ongoing coalition building				Studies and reports: Experiencing Cancer in Appalachian Kentucky and other investigations on cancer care and connectivity
**Define**								
	Define or redefine the problem to be solved. Align with all stakeholders on problem prioritization	La Jolla, California (November 13, 2018)		Tactical meeting dedicated to reviewing year 1 progress Concretize objectives				Blueprint for year 2 of LAUNCH
**Ideate**								
	Generate ideas from diverse stakeholders to improve cancer experiences in rural Kentucky	Pikeville, Kentucky (August 27, 2018) Lexington, Kentucky (February 9, 2019) McKee, Kentucky (June 17, 2019)		Connected health community ideation studio at SOAR^e^ Summit Ideation studio at Markey Cancer Center Ideation studio at People’s Rural Telephone Cooperative				Impromptu video pitches by summit attendees for ideas to *help local patients with cancer* Four co-designed concepts with diverse stakeholders: a portable cancer resource hub, a digital patient navigator, a community sourcing tool, and a wraparound support ecosystem Seven co-designed innovation recipes for helping rural patients with cancer
**Refine**								
	Select best concepts for prototyping. Co-design specific concepts	Lexington, Kentucky (October 7-8, 2019)		Co-design studios with diverse stakeholders				Artistic representations and video pitches of co-designed categories, questions, scoring system, and communication methods for a new monitoring tool Artistic representations and video pitches of co-designed electronic interfaces and service models for the new cancer symptom monitoring tool
**Implement**								
	Develop working prototypes of co-designed concepts Iterate on prototypes	Toronto, Canada and La Jolla, California (October 7, 2019) Berlin, Germany (October 8, 2019)		Remote, real-time development of prototypes by expert designers and developers				Prototype of a paper-based distress monitoring tool: *You and Your Well-being* • Prototype of an electronic distress monitoring tool: *myPath* Prototype of a provider dashboard and service design for these new tools
**Test**								
	Test the usability of the prototypes Collect feedback on the prototypes	Lexington, Kentucky (October 9-10, 2019)		User-feedback booths in cancer center lobby and at Markey Cancer Center Affiliate Network Annual Meeting				Usability surveys for paper-based tool, electronic tool, and provider dashboard Informal conversations and feedback about prototypes

^a^GIS: geographic information system.

^b^FCC: Federal Communications Commission.

^c^NCI: National Cancer Institute.

^d^C2H: Connect2HealthFCC Task Force.

^e^SOAR: Shaping Our Appalachian Region.

### Identify, Discover, Define

#### The Changing Face of Cancer Care

Cancer treatments are under constant innovation. Some examples include the recent development of minimally invasive surgical techniques, stereotactic radiation therapy, evolving immunotherapies, and the expanding field of precision oncology. However, although innovation in cancer treatment has drawn attention and resources, mechanisms for delivery across the continuum of diagnosis, staging, treatment, survivorship, and end of life have not kept pace [24,25]. Care delivery remains rooted in outpatient clinic visits to assess symptoms and provide results, requiring patients and caregivers to travel and take time off from work and family schedules. The logic of current care delivery is to move patients to information rather than information to patients. This can cause delays because patients must learn to navigate a complex care environment to obtain test results, fill prescriptions, and coordinate referrals to allied health services.

Symptom management, as well, has not kept pace. As advances in cancer treatment over the last decade have produced significant improvements in survival rates and significant reduction of morbidity [26,27], people with cancer now face the challenge of managing their disease as a chronic condition, often with distressing symptoms secondary to the disease process or treatment effects. Much of the burden of this symptom management falls on the person with illness and caregivers outside of the health care system [28].

Furthermore, the burdens of seeking care and managing symptoms are not equally distributed. People in traditionally underserved and rural communities are especially affected by limited access to essential resources. Geographic isolation, poverty, and transportation challenges, coupled with limited or no internet connectivity, can impede access to necessary support services [11,29,30]. Even in cases where broadband and internet connectivity are available, the challenges of rugged geography or long distances can cause connections to be unreliable and unstable. In these cases, novel, innovative methods may be required to ensure connectivity for critical health-related functions [31,32].

It is in this context of the changing landscape of cancer care and emerging technological possibilities that we identified an opportunity at the intersection of health and connectivity. Discovery proceeded with 6 weeks of ethnographic fieldwork conducted in communities and health care facilities across Appalachian Kentucky [12]. We sought to understand the experiences of local patients with cancer, cancer survivors, caregivers, and health care and broadband providers to inform problem definition and co-design strategies. We further sought to document the cancer care and connectivity infrastructure available in Appalachian Kentucky to build upon and integrate with existing resources as well as to learn from the shortcomings of previous solutions.

#### Definitions of Distress

In particular, our ethnographic work helped us to critically examine our approach to *distress*, a term common in the cancer symptom management clinical lexicon but which proved uncommon among those who had actually experienced cancer. However, our participants did speak extensively about the physical and psychosocial impacts associated with cancer and pointed to ways they coped with them.

According to the National Comprehensive Cancer Network (NCCN) Guidelines for Distress Management, *distress* is defined as a multifactorial unpleasant experience of a psychological (ie, cognitive, behavioral, and emotional), social, spiritual, and/or physical nature that may interfere with the ability to cope effectively with cancer, its physical symptoms, and its treatment [33]. Identification and treatment of distress have been shown to be critically important to improving health outcomes, quality of life, and adherence to recommended treatments. [34]. For this reason, distress screening at the time of cancer diagnosis is the recommended standard of care, and most cancer centers have shown improvements in routine screening [35].

Barriers to collecting these data have typically been related to workflow in the outpatient clinical setting. In addition, communicating results of patient-reported outcomes to providers has been a challenge, decreasing the impact of the information. Finally, collecting this sensitive and timely information from patients in the setting of the waiting room or during triage for a clinic visit may leave patients reluctant to share their actual feelings or symptoms, both past and present. In spite of these obstacles, the Commission on Cancer continues to emphasize the importance of distress screening as an important facet of patient care [36]. Commentators have been advocating for the use of implementation science methods, including those associated with HCD, to improve the effectiveness, reliability, and equity of distress screening efforts [37].

Our ethnographic findings indicated that people in Appalachian Kentucky ascribe different values to personal versus social suffering. Although personal suffering was considered taboo because it could cause others to suffer, suffering on behalf of another person was a source of pride because it indicated the strength of familial and community relationships. We also found that participants preferred to speak about this suffering in colloquial rather than clinical language, both to minimize the taboo of their personal suffering and to capture some nuance lacking in clinical terminology. As the LAUNCH stakeholders began to align on tackling the specific problem of distress monitoring and symptom management, we also began to align on more flexible definitions of key terminology and on a method for integrating the diverse perspectives of patients, caregivers, and clinicians in problem definition.

### Ideate, Refine, and Implement

**Figure 2:**
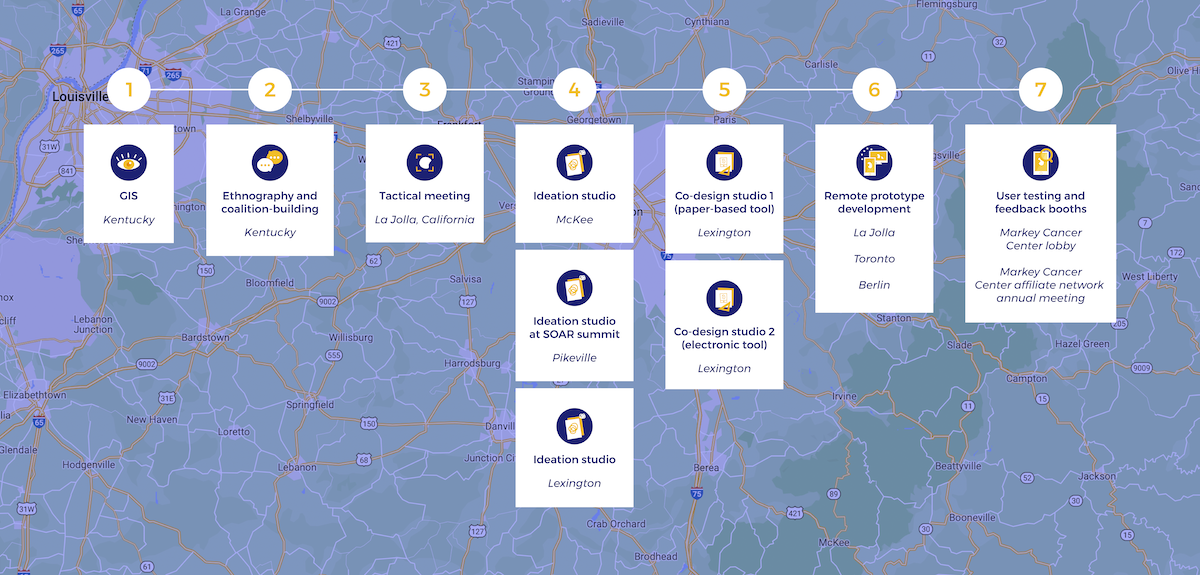
LAUNCH (Linking and Amplifying User-Centered Networks through Connected Health) co-design approach in rural Kentucky. The co-design, cocreation, and testing of the myPath system visualized as staged studios in Kentucky. (1) The initial problem statement counties with double burden of high cancer rates and low connectivity derived from geographic information system (GIS) observations across the United States. (2) Contextual inquiry and coalition building in the region was performed to obtain context, refine the problem statement, and develop a network of stakeholders. (3) Design studio 1 in Lexington focused on problem refinement and early discovery. (4) These findings were presented to a new group in McKee to ideate and generate low-fidelity concepts (solutions). (5) These concepts were refined in studio 3 to generate a single service design and midfidelity prototype. (6) In studio 5, high-fidelity, working prototypes were developed in a 2-day sprint. (7) These prototypes were tested in a concurrent, multisite 2-day usability pilot with diverse stakeholders. GIS: geographic information system; SOAR: Shaping Our Appalachian Region.

Methods for ideation, co-design, and development of low-fidelity prototypes were adapted from the IDEO Human Centered Design Toolkit [38] and used in LAUNCH Roadmap activities 4-6 (Figures 1 and 2 and Table 1). Ideation (activity 4) occurred over the course of 3 events in Kentucky described below. Refinement of concepts and development of prototypes (activities 5 and 6) were conducted over the course of a 4-day sprint in Lexington. Prototypes were first described verbally, then *developed* with low-fidelity methods such as Post-it Notes (3M) as well as whiteboards and corkboards. In parallel, a professional designer (in Toronto, Ontario, Canada), a mobile app developer (in Berlin, Germany), and a design support group (in La Jolla, California) worked as a *pair design* [39] distributed group to translate the low-fidelity concepts into working prototypes in 72 hours. These working prototypes were then iterated upon, in real time, by participants in Kentucky using web-based collaborative software. Further material can be viewed in the Multimedia Appendices 1-5.

### Test

#### Overview

Usability testing (activity 7) was conducted over the course of 2 days at booths at 2 separate locations. A booth was set up at the outpatient clinic area of University of Kentucky Markey Cancer Center (MCC) to collect feedback from a convenience sample, including patients, caregivers, and providers. Another booth was set up during the MCC Affiliate Network (MCCAN) Annual Meeting at the Lexington Convention Center held on October 9-10, 2019. The MCCAN Annual Meeting was attended by more than 300 cancer providers and researchers from across Kentucky and provided an excellent venue to collect feedback from a knowledgeable pool of attendees. Participants who came to these 2 booths were introduced to the paper versions of the instruments first, followed by the app versions. Providers and researchers were also invited to evaluate a digital dashboard developed specifically for providers receiving myPath data. Participants were given an opportunity to use these prototypes, and at the end, to complete a usability questionnaire to evaluate the respective prototypes against the gold standard, the paper-based NCCN Distress Thermometer (DT).

#### Survey Instrument

In all, 3 usability surveys were deployed in the usability testing booths. The first survey evaluated the paper versions of the two instruments (NCCN DT vs myPath); the second survey evaluated the digital versions of the 2 instruments; and the third survey evaluated the web-based myPath provider dashboard. In addition to collecting basic demographic data, we used the System Usability Scale (SUS) [40,41] to measure perceived usability and learnability of the standard instrument and co-designed prototypes.

#### Data Analysis

Descriptive statistics were reported. Independent 2-tailed *t* tests were used to compare the app version of myPath with the paper version of the NCCN DT and test the app version of either instrument versus the paper version of the same instrument. Paired *t* tests were used to compare the SUS scores of the NCCN DT versus myPath in the same format (either the paper or app version). A 1-way analysis of variance was conducted to compare mean SUS scores among different types of users. Results in multiple post hoc comparisons were adjusted using the Šidák method [42]. Analyses were performed in SPSS software (version 27; IBM Corp).

### Ethics Approval

This study received ethics approval from the University of California, San Diego institutional review board (record number 180589).

## Results

### Kentucky Co-design

We planned and carried out a sequence of community-based, participatory activities in Kentucky (Figure 2). (1-3) Framing and contextual inquiry set the stage for participatory design work. (4) Ideation began in Pikeville, then coalesced at an ideation studio in Lexington, which engaged patients, providers, and caregivers to frame problems in cancer care and conceptualize new strategies to solve these problems. This work contextualized the next design studio in an Appalachian county (Jackson) where community members, patients, and caregivers were encouraged to brainstorm granular solutions to problems generated in the Lexington studio or to make practical those solutions that had been considered. (5) These concepts were presented at a subsequent co-design sprint in Lexington to select a lead candidate. (6) We then prototyped and refined this concept and rapidly developed working interventions to *test* in the real world with patients, providers, caregivers, and others. (7) The outcome of this work was a new software application and service model developed with participants and delivered to stakeholders for implementation and formal testing.

### Outcomes

#### Overview

We have summarized the key outcomes of our design process in Table 1, showing the progression of the co-design activities. In the following sections, we detail the key findings of the refine, implement, and test activities in which we developed paper-based and digital prototypes and conducted usability testing of the 2 tools.

#### Paper-Based Prototype Development

**Figure 3:**
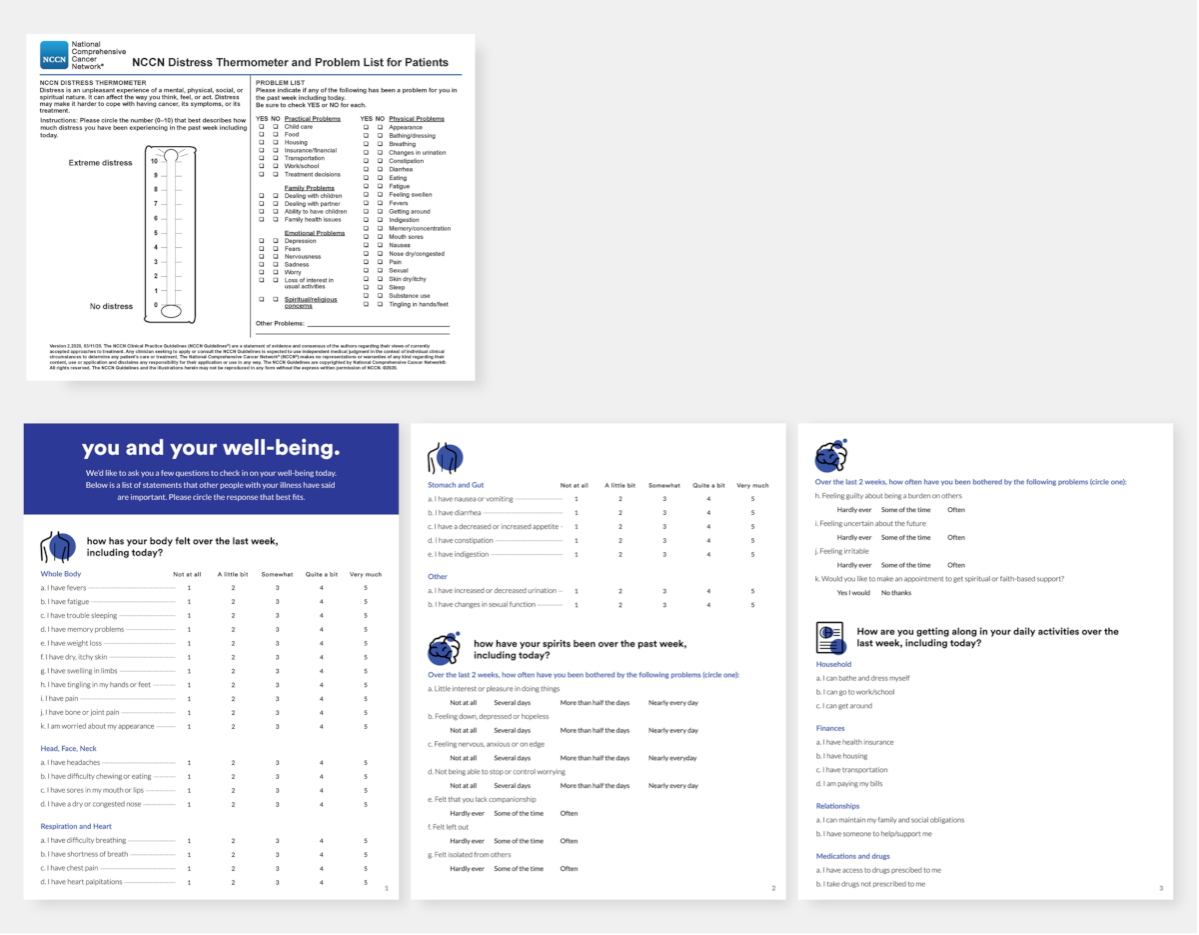
Comparison of paper prototypes. The gold standard National Comprehensive Cancer Network (NCCN) Distress Thermometer is shown (top) with the newly designed paper instrument developed to track patient progress and well-being.

As the existing NCCN tool is paper-based, at our design studio on October 7, 2019, we first developed a comparable paper tool inspired by co-design and cocreation work from prior studios. Key findings from these sessions (Table 1) produced the following requirements (and potential pitfalls of this approach): (1) compelling user experience, (2) framing the problem from patient perspective (*my wellness* and *my path* vs *Distress Thermometer*), (3) framing questions positively, (4) adding patient-centered questions, and (5) giving feedback and actionable information upon completion of the survey. The finalized design of the newly developed instrument is shown in comparison with the current accepted approach (NCCN DT) in Figure 3.

#### Digital Prototype Development

**Figure 4:**
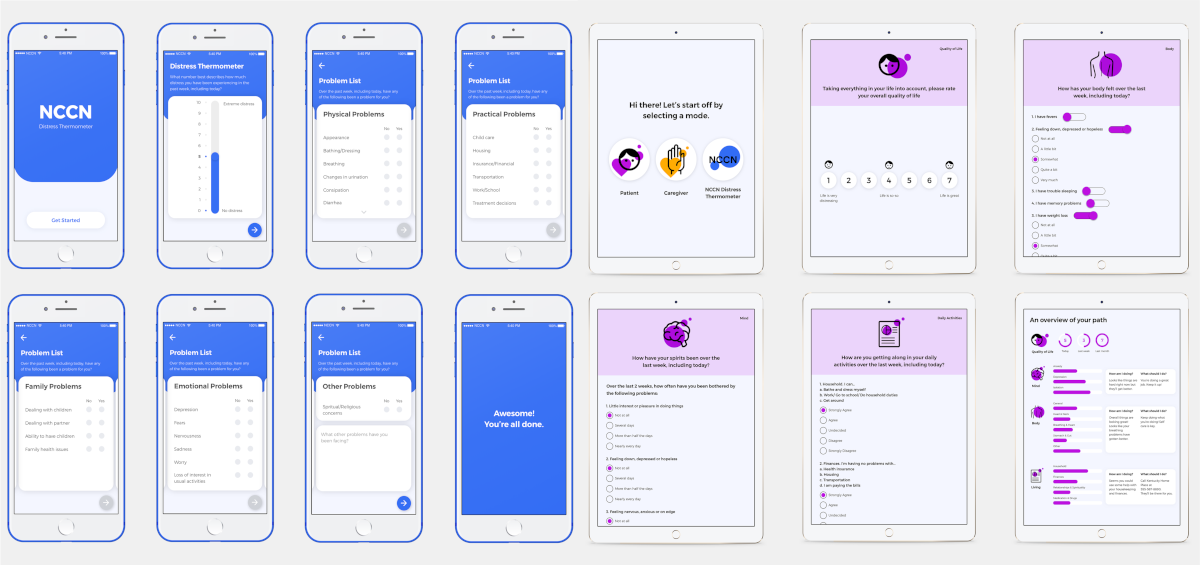
Comparison of *digital* distress thermometer (left) and myPath app (right).

Certain feedback from the design studios could not be implemented using paper-based methods alone and required digitization, for example, to get feedback based on answers and to communicate patient distress with providers in a timely manner. In the Lexington co-design studio on October 8, 2019, we worked with participants to co-design a digital version of the new paper tool (named myPath by the participants) as well as a digital version of the NCCN DT (Digital DT [DDT]) not only to assess incremental improvement from added functionality, but also to discern the role of digitization alone. Highlighted user requirements for myPath included compelling colors, content matching the new paper tool, new summary screen giving immediate feedback upon completion, and actionable insights with instructions and connection to providers. If patient-reported symptoms or needs through the myPath mobile app are over a predetermined threshold, an email alert will be sent to the care team and patients’ reports will be highlighted on the dashboard for providers to review. The care team will then decide the best approach to intervene. The developed DDT and myPath app are shown in Figure 4.

**Figure 5:**
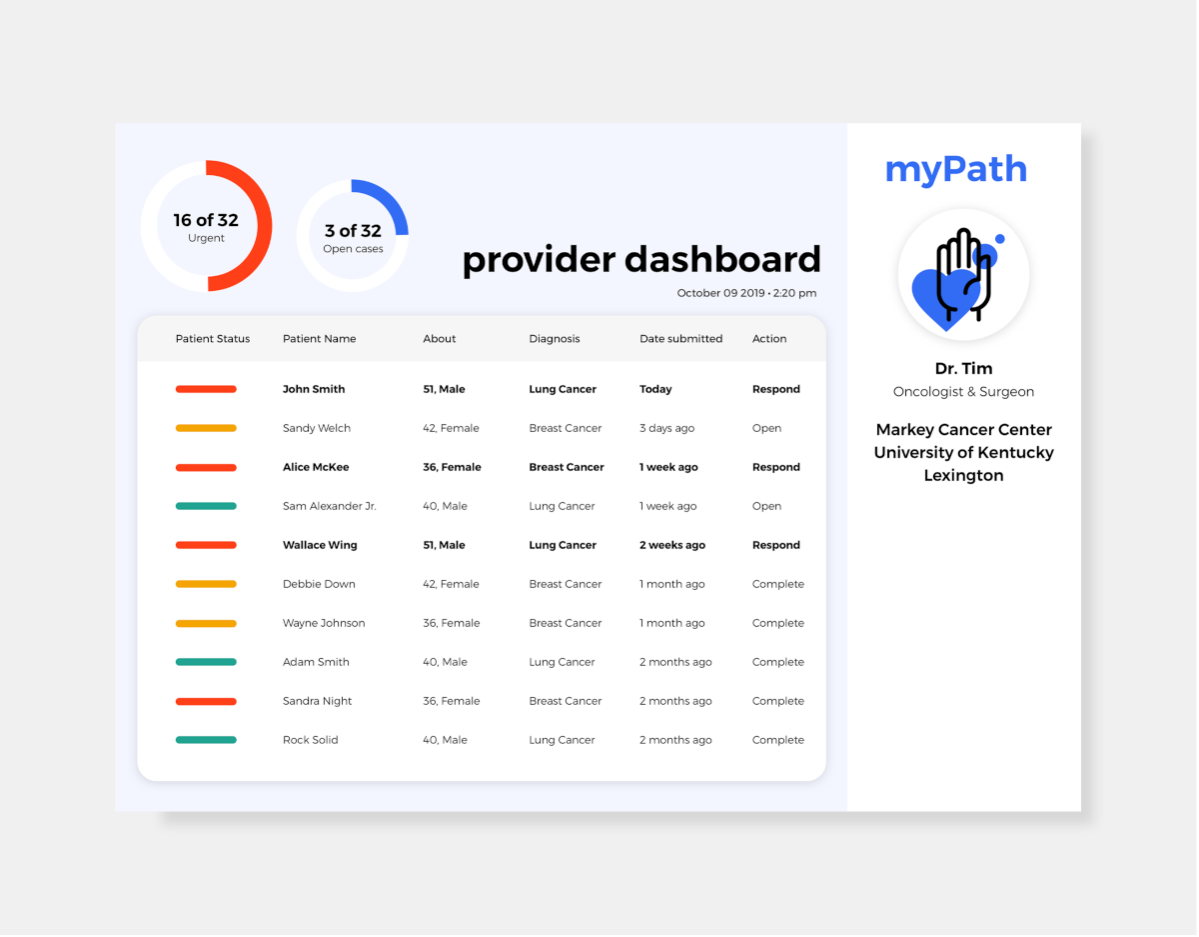
Clinical dashboard prototype.

Recognizing the other side of the patient–provider dyad, we prototyped a provider-centered dashboard meant to integrate with user-generated data in the myPath app. The key requirements for the dashboard as developed by the participating providers were as follows: (1) make it easy and clear to read, (2) only show critical information, (3) make it clear what has been done or what needs to be done, and (4) minimize disruption to provider workflows. The developed dashboard is shown in Figure 5.

### Prototype Testing

#### Overview

On both days (October 9 and 10, 2019) and at both locations (MCC clinics and MCCAN Annual Meeting), 86 participants completed a usability survey. Of the 86 participants, 46 (53%) evaluated the paper versions of both instruments of the NCCN DT and myPath, 34 (40%) reviewed the app versions, and 6 (7%) assessed the myPath dashboard. After removing surveys with missing data in the SUS questions, of the 86 participants, 38 (44%) evaluated the paper versions. Of these 38 participants, 5 (13%) were patients with cancer, 8 (21%) were caregivers, 11 (29%) were providers, there were 12 (32%) others (eg, technologists, payers, and service providers), and there were 2 (5%) with missing data on this question. Of the 27 participants who evaluated the app versions, 7 (26%) were patients with cancer or cancer survivors, 4 (15%) were caregivers, 8 (30%) were providers, and there were 8 (30%) others. Of the 6 participants who evaluated the dashboard, 4 (67%) were providers and 2 (33%) were researchers. Overall, more than half of the participants were aged >50 years, and 96% (83/86) used a smartphone. Among the 83 smartphone users, 64 (77%) had an iPhone.

**Figure 6:**
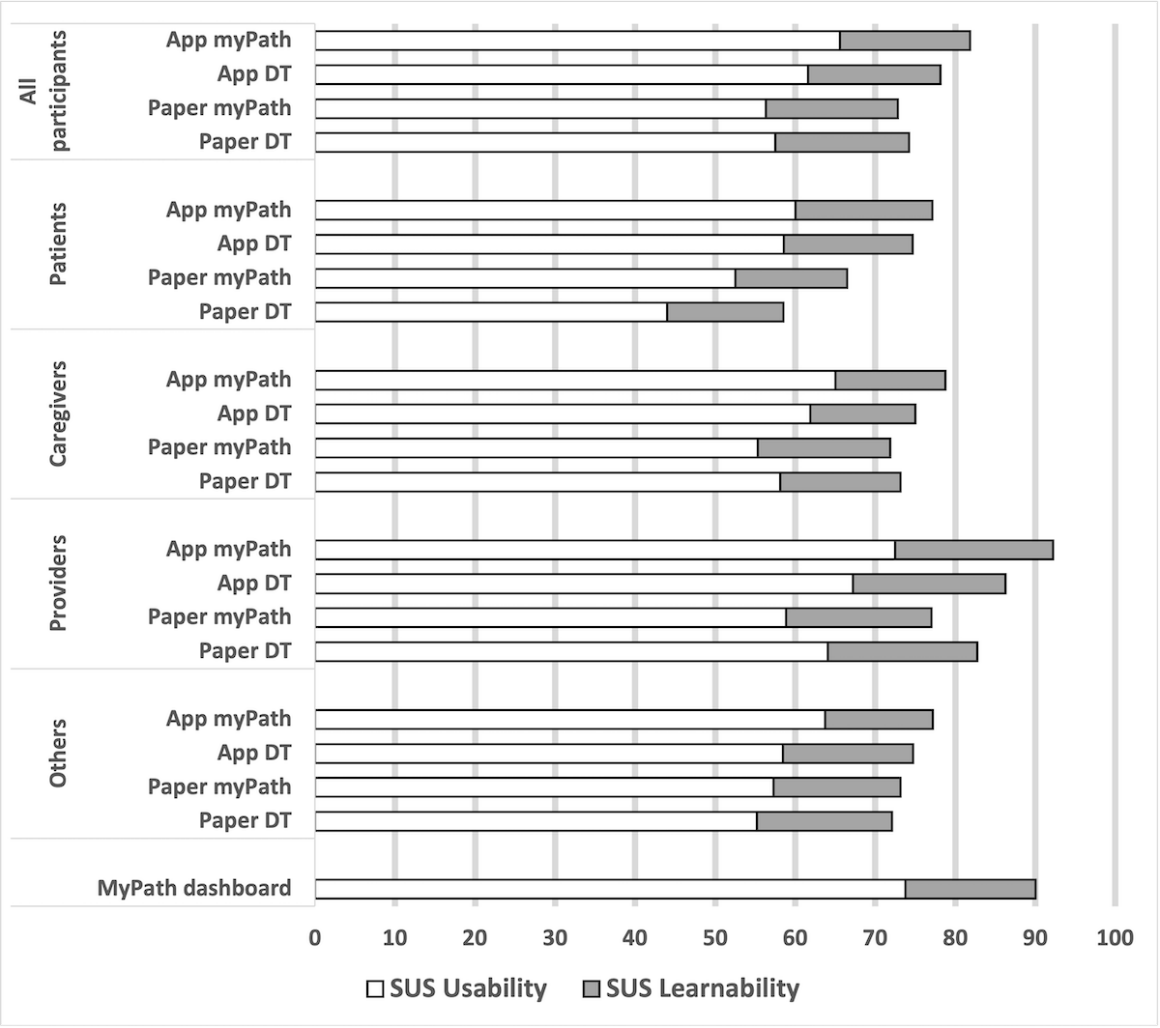
Stacked bar chart of System Usability Scale (SUS) subscales. DT: Distress Thermometer.

Figure 6 shows the results of usability surveys with participants, plotted by participant and prototype assessed. We show the sum and 2 SUS subscales for the digital myPath (App myPath), the NCCN DDT (App DT), the paper prototype of myPath (Paper myPath), NCCN DT (Paper DT), and the provider dashboard. The error bars represent the SDs of the subscales. On average, the overall SUS scores of both instruments for both the paper and app versions as well as the dashboard are higher than a cutoff value at 68-70, showing that both instruments, in either format, have above-average usability [41,43]. Specific comparisons, including statistical testing results, are presented in the next sections. However, because this study was not designed or powered to detect the differences among instruments, the comparative results reported here should be considered preliminary evidence.

#### myPath App Versus Paper DT

Across participants, the digital myPath app showed the highest perceived combined usability (mean 81.9, SD 15.2) compared with the current gold standard of distress management for patients with cancer, the Paper NCCC DT (mean 74.2, SD 15.8). Testing of the SUS subscales showed that the myPath app had significantly better usability than the Paper DT (t_63_=2.611; *P*=.01; Cohen *d*=0.657, 95% CI 0.148-1.161), whereas the learnability did not differ between the instruments (t_63_=–0.311; *P*=.76; Cohen *d*=–0.078, 95% CI –0.571 to 0.416).

#### App Versus Paper Version of the Same Instrument

To discern differences that could be attributed to content versus digitization, we compared paper and digital approaches (Figure 6). Here, the digital version of myPath showed a significantly higher overall SUS score than the paper version (t_63_=2.345; *P*=.02; Cohen *d*=0.59, 95% CI 0.084-1.092), and this difference was significant for the usability scale (t_63_=2.991; *P*=.004; Cohen *d*=0.753, 95% CI 0.24-1.261), although not for the learnability scale (t_63_=–0.157; *P*=.88; Cohen *d*=–0.04, 95% CI –0.533 to 0.454). However, the difference between the DDT and Paper DT was not statistically significant (*P*=.34 and *P*=.24, respectively). Across participant roles, providers (mean 92.19, SD 7.61) reported significantly higher SUS scores (t_17_=3.164; *P*=.006; Cohen *d*=1.47, 95% CI 0.419-2.488) for the myPath app than for the paper myPath (mean 77.05, SD 11.82). No significant difference was found when comparing providers’ ratings of DDT versus Paper DT (t_17_=0.567; *P*=.58; Cohen *d*=0.263, 95% CI –0.655 to 1.174).

#### myPath Versus NCCN DT in the Same Format

The paper myPath tool had a slightly lower overall SUS score (mean 72.8, SD 15.3) than the paper DT (mean 74.2, SD 15.8). In contrast, the myPath app (mean 81.9, SD 15.2) had a slightly higher SUS score than the DDT (mean 78.1, SD 17.1). However, these differences were not statistically significant (*P*=.60 and *P*=.20, respectively). These comparisons were also not statistically different in the 2 SUS subscales.

#### Patients’ Versus Providers’ Ratings of the Same Instrument

Compared with patients, providers reported higher SUS scores for all instruments (Figure 6). In the 1-way analysis of variance, the overall SUS score for the Paper DT differed significantly between participant roles (*F*_3,32_=3.28; *P*=.03). Specifically, patients reported a significantly lower overall SUS score than providers (Cohen *d*=–24.23, SE=7.85; *P*=.03; 95% CI –46.24 to –2.21). This difference was mainly seen in the usability subscale (*F*_3,32_=3.21; *P*=.04) and not the learnability scale (*F*_3,32_=1.902; *P*=.15). The same test did not show significantly different SUS scores between participant roles for the paper myPath, DT, or myPath app.

## Discussion

### Summary of Results

In this study, we report the results of an ongoing effort to improve the resilience of an oncology system, and a health care system in general, that is struggling to provide equitable access to care for patients both within and outside of the clinical encounter. Specifically, we identified the problem of remote distress monitoring for patients with cancer as an emblematic first step by which a public–private partnership could demonstrate the value of connected cancer care through innovative design methods that democratize the development of solutions. In our efforts, we began with the recognition that successful implementation of connected health solutions in real-world settings requires more than providing broadband access to patients and their care teams. Such an approach requires a careful restructuring of the local workflows and communication channels needed to give patients and their providers the confidence to engage in proactive care irrespective of physical distance or scheduled appointments. Moreover, re-engineering workflows is not something that can be done successfully from the top down; adaptation must be driven by the very individuals who understand the local context. Moreover, this process must have testable, objective outcomes to drive iteration to a clear win state that recognizes both institutional and individual needs.

To facilitate this local adaptation, we created a design and implementation framework (Table 2) informed by our collective experience in human factors, cognitive engineering, anthropology, public health, health communication, design, epidemiology, and clinical medicine. The framework began with a problem-identification stage, which framed geographic areas for contextual inquiry through the combined triangulation of epidemiologic data from registries of cancer burden with industry-level databases on broadband access. The results of these quantitative studies were then used to guide further discovery, problem refinement, and ideation through ethnographic observations, interviews, and semistructured group meetings in those areas of Appalachian Kentucky struggling with the double burden of poor cancer outcomes and lack of access to broadband. These locally co-designed solutions were then prototyped, refined, and tested as part of an implementation solution in Appalachian Kentucky. Clinical trials are currently underway to test the efficacy of these solutions.

**Table 2:** LAUNCH (Linking and Amplifying User-Centered Networks through Connected Health) framework: lessons learned.

Step	Innovation	Lessons learned
Identify	GIS^a^ analysis: data sharing across organizations and analytic teams helped to identify double-burden regions	Calibration of parameters across data sets took time; once calibrated, the resulting geographic maps served to focus community efforts
Discover	Cognitive ethnography: used a blended methodology combining cognitively based protocol analysis (focusing on how individuals perceive, process, and use cancer information) with ethnographic techniques from medical anthropology designed to elucidate culture, norms, roles, and values	Cognitive inquiry proved to be useful for improving wording, formatting, and sequencing at the individual user level, whereas the ethnographic work helped address systemic implementation at the community level
Define	Tactical refinement: a high-level, cross-sector *think tank* was convened to review data and establish priorities in follow-up to information gathered during the discovery phase	Strategic discussions were most productive when they transcended misaligned incentives to identify mutually agreeable objectives across the full ecosystem of care within the targeted communities
Ideate and refine	Co-design: multi-stakeholder teams of clinical and community representatives worked in collaboration with technology developers to co-design an end-to-end system for monitoring and reducing patients’ distress	Co-design worked best when it followed a rapid sprint model for prototyping and then testing the key components needed to support productive interactions among patients, caregivers, and clinical teams
Implement	Clinical adaptation of reusable components: the LAUNCH development process yields a reusable library of technologies and protocols, which can then be adapted and evaluated locally within functional systems	Effective clinical care within communities requires adaptation to customize telemedical components using the resources, infrastructure, and people available within local ecosystems of care linked through accountable data structures
Test	LAUNCH-PAD^b^: a platform that allows for pragmatic assembly and testing of crucial components safely within clinical settings	The implementation science needed to customize service structures in a timely and responsive fashion should adhere to pragmatic trial evaluation approaches

^a^GIS: geographic information system.

^b^PAD: Platform for Agile Development.

Our goal in publishing these results at this time is to give an indication of how use of the framework could help inform design decisions from the ground up as communities adapt to the complex interplay of remote telemedical options and in-person consultation in a period of intense change in health care. In our case, the ethnographic work we conducted in collaboration with MCC and MCCAN highlighted a need to adapt the traditional verbiage and approach of the NCCN-mandated stress measures to improve buy-in (adoption) and increase comprehension, consider a wellness frame (as opposed to a sick frame), and increase patient and provider engagement. This is just one aspect of system redesign that may often be overlooked without careful examination of patients’ (people’s) values and predilections in the geographic regions in which they are served [16]. Once these were identified, we engaged in co-design efforts with these groups to improve how the instrument could address community prioritized needs in a manner that is locally acceptable, effective, and sustainable.

The results from our iterative evaluation process showed incremental improvements in usability and learnability among patients as we progressed through our iterative development path from the paper-based NCCN DT to a reworded version of the paper-based instrument to a digital adaptation of the NCCN DT and finally to an electronic adaptation of the myPath app. Providers, on the other hand, seemed to be less comfortable with the earlier iteration of the paper-based myPath tool than patients. This seemed to be because clinical staff were more familiar with the traditional language in the NCCN DT and thus felt more comfortable with the existing verbiage. Nevertheless, providers responded with even higher usability and learnability ratings than patients when exposed to the final electronic version of the myPath app. Providers also offered extremely high ratings for the myPath dashboard. From our experience, it seems that the clinical staff grew more appreciative of the form and intent of the project the closer it progressed to its final operable format. The oncology teams were especially taken with the enhanced capacity the dashboard offered in management of caseloads across patients.

### Implications

When we began our efforts, we relied on an evolving evidence base in cancer care suggesting that strategic deployment of remote, *point-of-need* technologies can offer instrumental gains for improving medical outcomes, protecting quality of life from the burden and cost of travel to appointments, and for offering intervention opportunities well before emergency services may be needed. In other words, we were following a typical evolutionary path for implementation in cancer care; one that—just like telemedicine more generally—would take years to complete.

Since then, we have watched as the necessities for physical distancing during the COVID-19 pandemic have pushed policy makers and health care administrators to move more rapidly on making connected care an integral part of 21st century medicine. Changes have included provisions for reimbursement by the Centers for Medicare & Medicaid Services, a relaxation of jurisdictional barriers across state lines, a softening of privacy restrictions under the Health Insurance Portability and Accountability Act, and the provision of financial incentives for building out broadband support for medicine within underresourced communities. It is unclear at this time how much of this policy change will continue after the pandemic subsides. What is clear is that a focus on HCD will be crucial as we iterate forward toward new service models in a rapidly changing and continuously challenging time. What is also clear is that these changes in the medical landscape will go well beyond the benefits they may convey to cancer care; they will be applicable to all other facets of care as we move to create an antifragile system for patients and the professionals who care for them.

### Strengths, Limitations, and Future Direction

This paper presents a framework for guiding HCD activities at the community level. It also offers insight into the process of applying the framework to the specific objective of improving distress monitoring processes for patients with cancer living in rural areas surrounding MCC in Lexington. As noted earlier, these efforts are part of the LAUNCH initiative [11]; therefore, it is only one part of a larger, unfolding story. Clinical pilot studies are currently underway to gauge the overall impact that our HCD efforts are expected to have on system and patient metrics. The true test of a community-based HCD approach will not be completely evident until much farther down the line.

Another limitation of this study is that it offers only one example of how a community-driven design approach could be applied to meet the needs of an academic medical center serving a largely rural catchment area. The way to scale the approach, we believe, is to offer pathways by which other communities around the country could apply best practices in HCD to their own local jurisdictions following a true platform-based model of deployment [44]. Future efforts are planned to create a *Platform for Agile Development* (ie, *LAUNCH-PAD*) to facilitate local resource matching, knowledge transfer, and a guided application of HCD resources and data within the local context.

### Conclusions

In this work, we described the implementation and testing of a co-design framework developed to address a global need for rapid health system innovation that generates effective and locally sustainable solutions that can scale laterally to promote resilience in the system. We hypothesized that this antifragile approach, bridging lead-user innovation with equity, diversity, and inclusion through co-design, could promote resilience in rapidly changing and increasingly uncertain times. Through this process we showed that codeveloped solutions addressing community-defined problems could be produced quickly, with broad stakeholder input, balancing goal-oriented, time-limited development with open and inclusive dialog. In conclusion, we encourage bringing together people with lived expertise and diverse and even dissimilar views because this creates the circumstances and creative outputs required for resilient, scalable, and locally acceptable solutions.

## Appendix

Multimedia Appendix 1 Think tank overview.

Multimedia Appendix 2 Deep Dive 1: empower people.

Multimedia Appendix 3 Deep Dive 2: activate communities.

Multimedia Appendix 4 Deep Dive 3: pay for value.

Multimedia Appendix 5 Kentucky Design Sprint in pictures.

## Abbreviations

DDT: Digital Distress Thermometer
DT: Distress Thermometer
HCD: human-centered design
LAUNCH: Linking and Amplifying User-Centered Networks through Connected Health
MCC: Markey Cancer Center
MCCAN: Markey Cancer Center Affiliate Network
NCCN: National Comprehensive Cancer Network
SUS: System Usability Scale
