# Supplementary figures and images for: Designing a Framework for Remote Cancer Care Through Community Co-design: Participatory Development Study
Source: J Med Internet Res. 2022 Apr 12;24(4):e29492. doi: 10.2196/29492 (PMC9044168; doi:10.2196/29492)

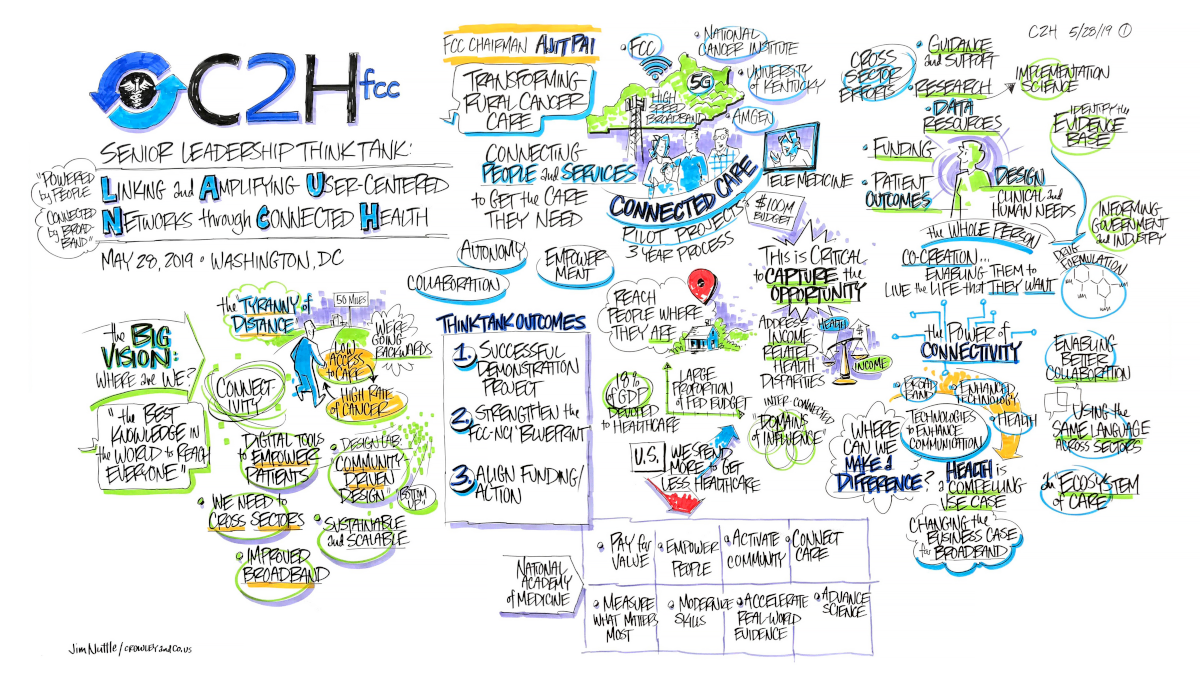

Supplement: Multimedia Appendix 1 [file jmir_v24i4e29492_app1.png]

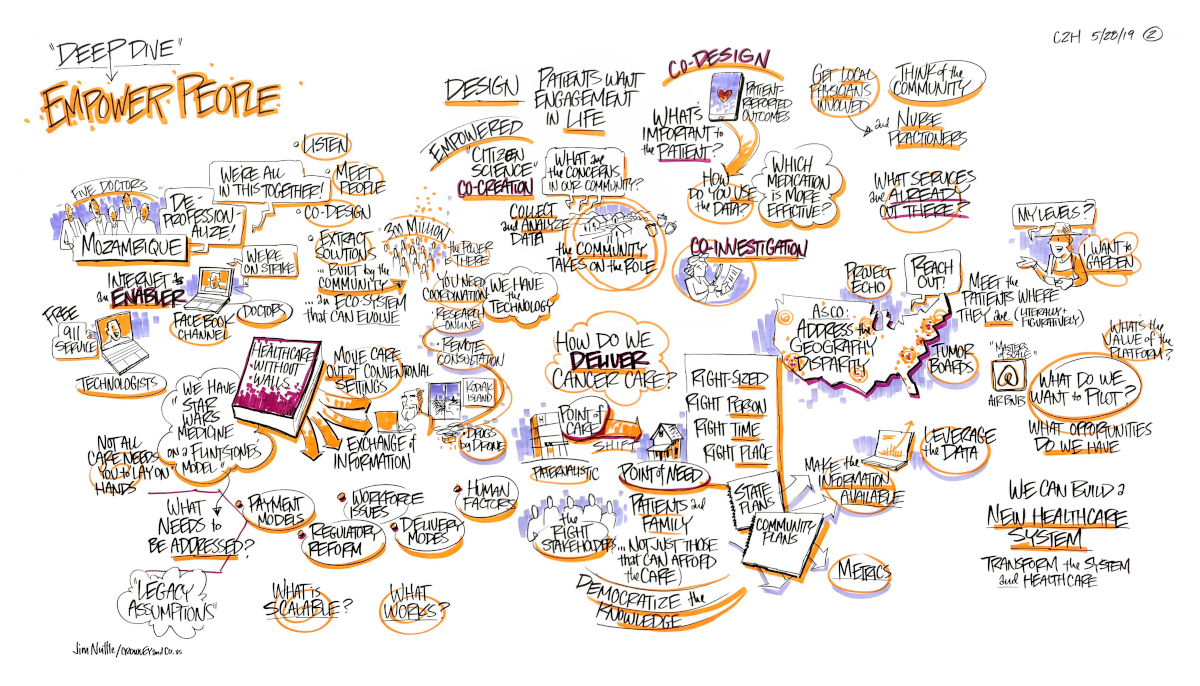

Supplement: Multimedia Appendix 2 [file jmir_v24i4e29492_app2.png]

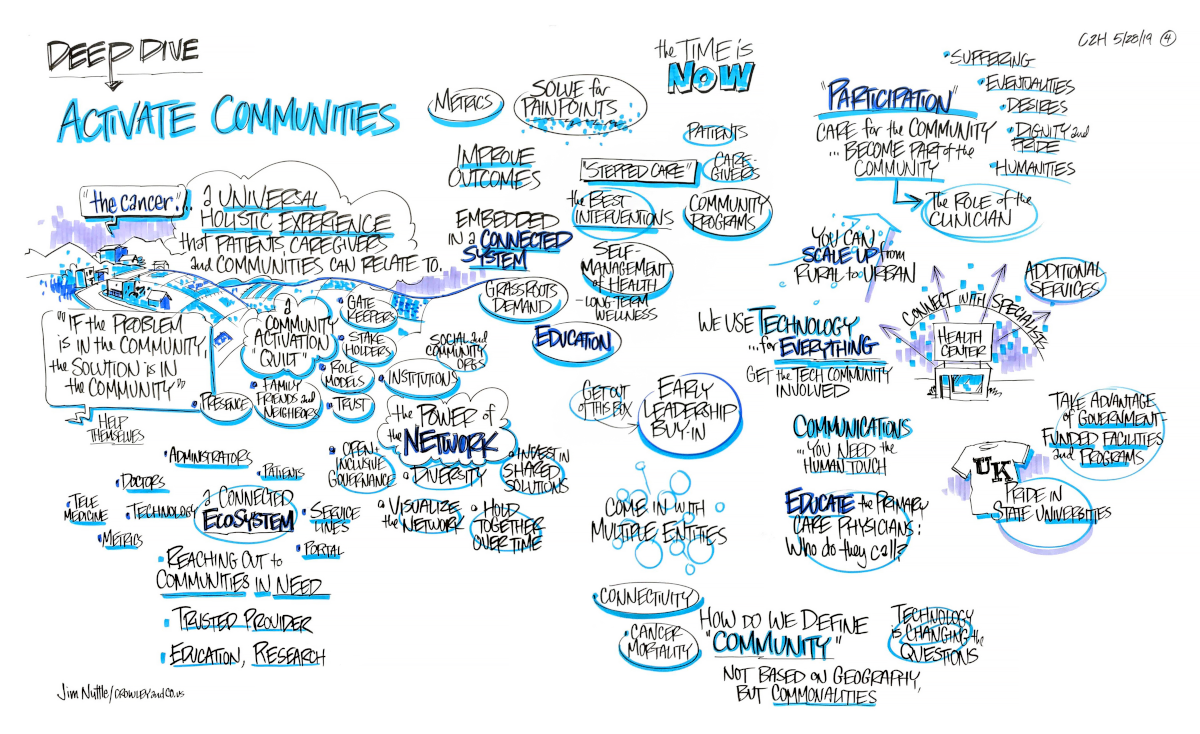

Supplement: Multimedia Appendix 3 [file jmir_v24i4e29492_app3.png]

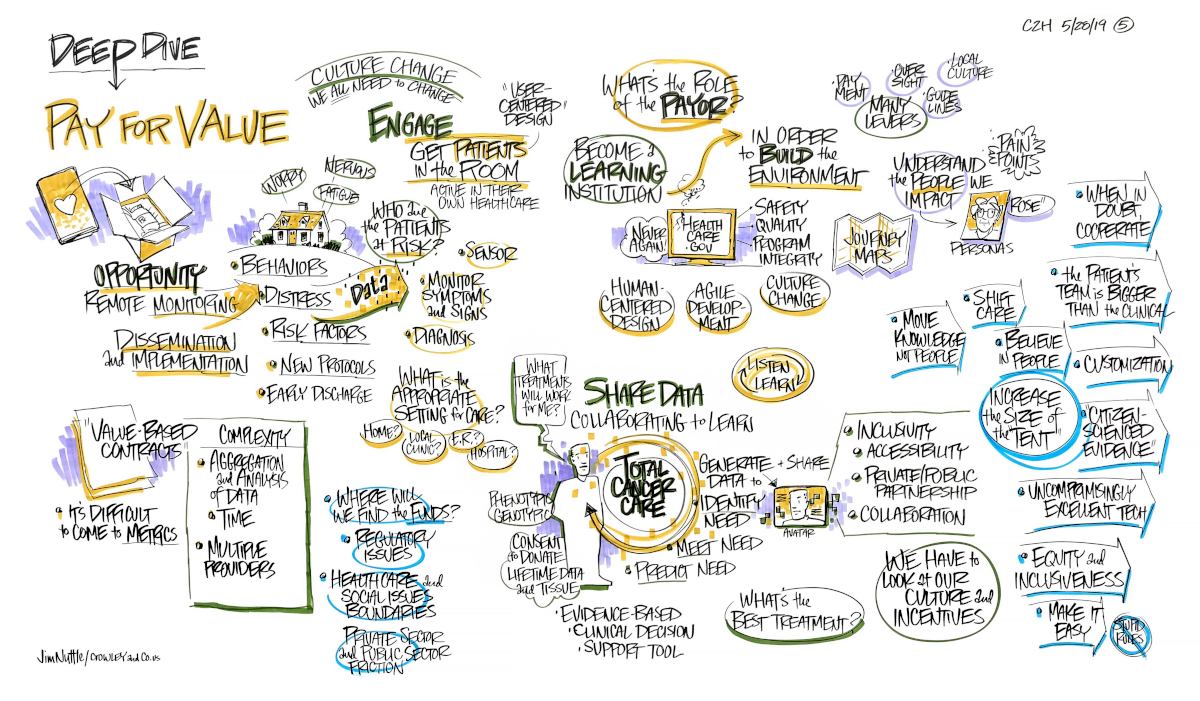

Supplement: Multimedia Appendix 4 [file jmir_v24i4e29492_app4.png]

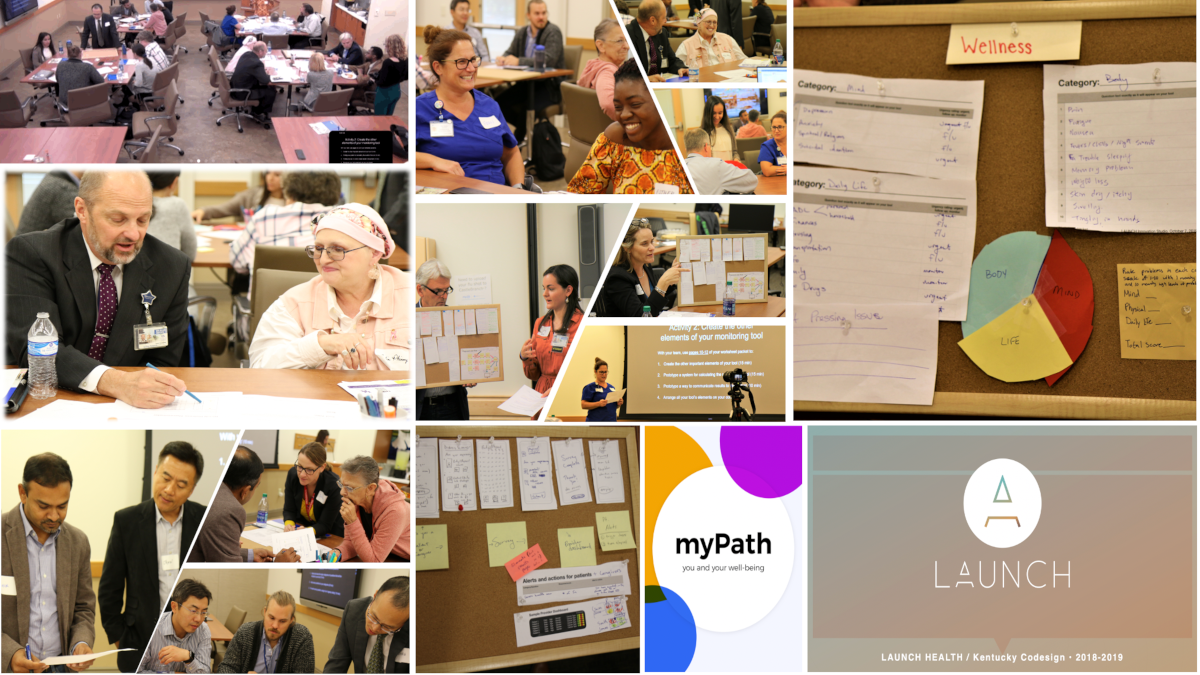

Supplement: Multimedia Appendix 5 [file jmir_v24i4e29492_app5.png]
